# Probing inhibition mechanisms of adenosine deaminase by using molecular dynamics simulations

**DOI:** 10.1371/journal.pone.0207234

**Published:** 2018-11-16

**Authors:** Xiaopian Tian, Ye Liu, Jingxuan Zhu, Zhengfei Yu, Jiarui Han, Yan Wang, Weiwei Han

**Affiliations:** 1 Key Laboratory for Molecular Enzymology and Engineering of the Ministry of Education, School of Life Science, Jilin University, Changchun, China; 2 Department of General Surgery, China-Japan Union Hospital of Jilin University, Changchun, China; Universidade Nova de Lisboa Instituto de Tecnologia Quimica e Biologica, PORTUGAL

## Abstract

Adenosine deaminase (ADA) catalyzes the deamination of adenosine, which is important in purine metabolism. ADA is ubiquitous to almost all human tissues, and ADA abnormalities have been reported in various diseases, including rheumatoid arthritis. ADA can be divided into two conformations based on the inhibitor that it binds to: open and closed forms. Here, we chose three ligands, namely, FR117016 (FR0), FR221647 (FR2) (open form), and HDPR (PRH, closed form), to investigate the inhibition mechanism of ADA and its effect on ADA through molecular dynamics simulations. In open forms, Egap and electrostatic potential (ESP) indicated that electron transfer might occur more easily in FR0 than in FR2. Binding free energy and hydrogen bond occupation revealed that the ADA-FR0 complex had a more stable structure than ADA-FR2. The probability of residues Pro159 to Lys171 of ADA-FR0 and ADA-FR2 to form a helix moderately increased compared with that in nonligated ADA. In comparison with FR0 and FR2 PRH could maintain ADA in a closed form to inhibit the function of ADA. The α7 helix (residues Thr57 to Ala73) of ADA in the closed form was mostly unfastened because of the effect of PRH. The number of H bonds and the relative superiority of the binding free energy indicated that the binding strength of PRH to ADA was significantly lower than that of an open inhibitor, thereby supporting the comparison of the inhibitory activities of the three ligands. Alanine scanning results showed that His17, Gly184, Asp295, and Asp296 exerted the greatest effects on protein energy, suggesting that they played crucial roles in binding to inhibitors. This study served as a theoretical basis for the development of new ADA inhibitors.

## 1. Introduction

Adenosine deaminase (ADA; EC 3.5.4.4) catalyzes the irreversible deamination of adenosine and 2′-deoxyadenosine to inosine and 2′-deoxyinosine, respectively. ADA is implicated in purine metabolism and distributed in most mammalian tissues [1]. Genetic ADA deficiency results in lymphopenia and severe combined immunodeficiency disease by decreasing the differentiation and maturation of lymphoid cells [2]. ADA is involved in T-cell activation [3]. Ecto-ADA can prolong chronic inflammation by degrading lymphocyte toxic extracellular adenosine or 2′-deoxyadenosine [4]. As an endogenous immunoregulator in cancer pathways, the role of adenosine in human brain functions and its potential value for clinical applications have been extensively investigated [5]. Increased ADA levels are associated with numerous diseases, and ADA inhibitors have been clinically used as antimetabolic and antineoplastic agents and modulators of neurological function because of their effects on adenosine levels [6].

**Fig 1:**
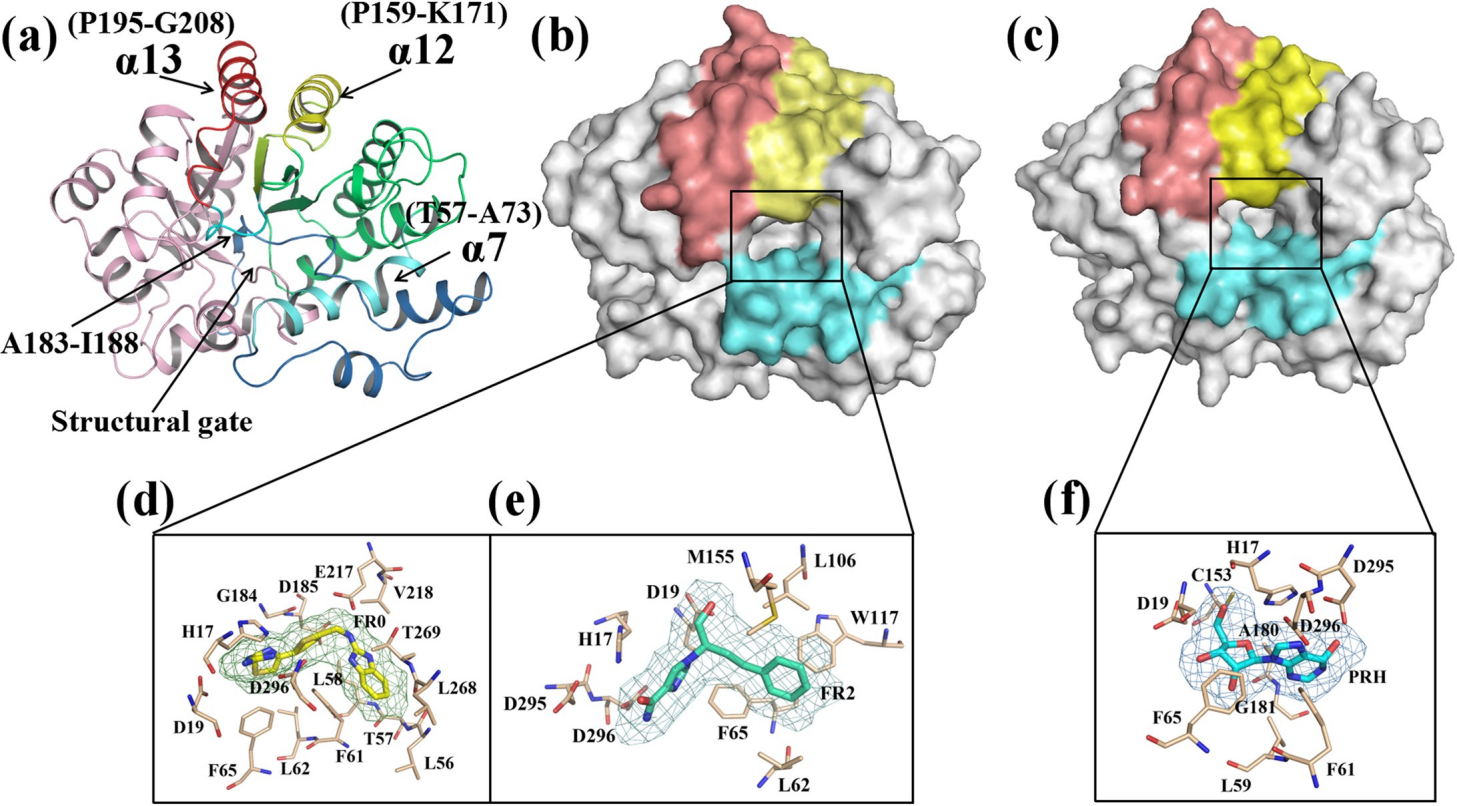
Overview of adenosine deaminase (ADA, PDB code: 1VFL) and the three ligands. (a) ADA structure. The α12 helix (P159-K171) is colored yellow; the α13 helix is colored red; and the structural gate is colored cyan, which consists of the α7 helix (T57-A73) and residues A183 to I188. (b) The binding pocket of ADA with an open form. (c) The binding pocket of ADA with a closed form. (d) The ligand FR0 (obtained from PDB: 1DNV). (e) The ligand FR2 (obtained from PDB: 1NDW) and (f) the ligand PRH (obtained from PDB: 1KRM) are denoted by sticks and mesh. The surrounding residues are depicted by light orange sticks.

X-ray crystallographic analysis revealed that ADA exhibits a triose phosphate isomerase-barrel conformation [7] (Fig 1A). This barrel consists of eight central β-strands and eight α-helixes [8]. ADA has two distinct conformations, namely, open and closed forms, which are observed in the examination of ligated states with different inhibitors [9]. In the closed form, the active site consists of hydrophilic S0 and hydrophobic F0. The S0 subsite, where the substrate adenosine mainly binds to, is completely enclosed by a structural gate consisting of the peptide backbone of a loop (Ala183 to Ile188) and two leucine side chains from an α-helix (Thr57-Ala73) [10]. In an open form, the gate is open. In comparison with closed forms, open forms have two additional hydrophobic subsites named F1 and F2 around the gate [11] (S1 Fig). The structural difference is due to an inhibitor-induced conformation change.

Many ADA inhibitors have been reported, and they can be classified conventionally into two types, namely, transition- and ground-state inhibitors [12]. Transition-state inhibitors of ADA are endowed with a structure that resembles a tetrahedral transition-state intermediate as a result of deamination. Ground-state inhibitors of ADA are characterized by a structure similar to that of adenosine. However, they are limited by a number of problems, such as toxicity [13], poor oral absorption, and rapid metabolism [14]. Therefore, a third class of derivatives, namely, non-nucleoside inhibitors, has been developed by employing a rational de novo design. The crystal structures of ADA with several nucleoside inhibitors serve as the basis for the design provided by Fujisawa Pharmaceutical Company [15]. Non-nucleoside inhibitors of ADA comprise a series of imidazole-4-carboxamides. The lead compound 1-(1-hydroxy-4-phenylbutan-2-yl)-1H-imidazole-4-carboxamide (FR117016, hereafter referred to as FR0) is characterized by a potent inhibitory effect and favorable pharmacokinetic properties. FR221647, another novel and unusual lead compound hereafter referred to as FR2, is characterized by a favorable inhibitory profile and an undesirable cytotoxicity.

To investigate the inhibitor-induced conformation change, we selected three inhibitors from the two types of ADA inhibitors, namely, FR0 (non-nucleoside inhibitor), FR2 (non-nucleoside inhibitor), and PRH (transition-state inhibitor), as the ligands of ADA (Fig 1B–1F). We used Gaussian09 [16] to optimize the structures of the three inhibitors. ADA presents an open form when it binds to FR0 and FR2 or a close form when it binds to PRH. Molecular dynamics (MD) simulation was performed to examine the structural changes induced by the binding of inhibitors. Principal component analysis (PCA) and free energy landscape (FEL) methods were applied to explore the functional dynamics and conformation changes in ADA. Linear interaction energy (LIE) method was employed to calculate the binding free energy. In this approach, the binding free energy was estimated from the change in the electrostatic (ele) and van der Waals (vdW) interaction energy between the ligand and its environment upon complex formation. Our study revealed the conformational changes associated with open and closed forms and might be valuable for future works.

## 2. Materials and methods

### 2.1 Preparation of initial complexes

In this study, we established five systems (S1 Table), namely, ADA, ADA-FR0, ADA-FR2, ADA-PRH, and ADA (without PRH). The crystal structures of the first four systems were retrieved from the Protein Data Bank (PDB) [17], and their PDB codes were 1VFL [9], 1NDV [18], 1NDW [18], and 1KRM [19], respectively. Sequence alignment results (S2 Fig) indicated that single difference was located in residue 47 between 1KRM and the three other complexes. Therefore, we removed the ligand PRH from 1KRM and considered this protein as the fifth system named ADA (without PRH). In this way, we set two groups for the five systems. The first group included three systems, namely, ADA, ADA-FR0, and ADA-FR2, and the second group comprised two systems, namely, ADA-PRH and ADA (without PRH), which was obtained by removing the PRH from ADA. The structures of the three ligands were optimized at the B3LYP 6–31 G* level [20] by using Gaussian09 [16].

### 2.2 MD simulation

A Gromos 53a6 force field [21] was applied to proteins and ligands. The parameterization of the three ligands was produced by the PRODRG2.5 server [22]. All of the systems were subjected to MD simulation by using a SPC water model [23] in a periodic box. Chloride and sodium ions were added to a random place as a replacement for the water molecules in the simulation box to neutralize the systems. Energy minimization was performed using the steepest descent method to equilibrate the initial structure. Afterward, 100 ps NVT (constant number of particles, volume, and temperature) [24] and 100 ps NPT (constant number of particles, pressure, and temperature) [25] were adopted to stabilize the environment of the systems at 300 K and 1 bar. The coupling constant was set to 0.1 and 2.0 ps for temperature and pressure, respectively. Particle mesh Ewald algorithm [26] was used to describe the long-range electrostatic interactions. The vdW interactions were calculated using a cutoff of 1.4 nm. LINCS algorithm [27] was utilized to constrain all of the bond lengths. Finally, the MD simulations of the five systems were performed. The simulation lasted 100 ns with a time step of 2 fs, and all of the coordinates were saved every 2 ps [28]. The MD simulations were submitted twice.

### 2.3 Principal component analysis

Principal component analysis (PCA) can provide a brief description of the motions and help in the recognition and binding of biomolecular systems [29]. PCA extracts highly correlated fluctuations from MD trajectories by applying dimensional reduction [30]. PCA is defined on the basis of the calculation and diagonalization of the covariance matrix to obtain orthogonal eigenvectors and corresponding eigenvalues. In this covariance matrix, Cij is defined as follows [31]:

$$C_{ij}=\frac{\langle \Delta r_{i}\cdot \Delta r_{j}\rangle }{(\langle \Delta r_{i}{}^{2}\rangle \cdot {\langle \Delta r_{j}{}^{2}\rangle )}^{1/2}}$$ (1)

where i (j) is the ith (jth) atom (or residue), and Δri (Δrj) is the displacement vector corresponding to the ith (jth) atom of the systems, indicating an ensemble average. The eigenvectors of the matrix represent the directions of the concerted motions, whereas the eigenvalues of the matrix indicate the magnitude of the motions along the direction. The first few PCs are the eigenvectors with the highest eigenvalues related to the functional motions of a biomolecular system [31]. In this article, PCA was performed using the GROMACS 5.1.4 package to investigate and compare the functional motions of the five systems.

The FEL [32] can explain the dynamic process occurring in a biological system [33]. In the FEL, the free energy minima represents the conformational ensembles in stable states, which are accessible to a biomolecule under physiological conditions. The first PCs can capture most of the important dynamics and can therefore be used to construct the FEL. The FEL can be estimated by

$$\Delta G\left(X\right)=-K_{B}T\ln P(X)$$ (2)

where KB is the Boltzmann constant, T is the temperature of the simulation, and P(X) is the probability distribution of the system along the reaction coordinate X. In our study, FEL was calculated to identify the dominant conformational states with relatively low energies of the five systems.

### 2.4 Linear interaction energy analysis

FEW [34] can be used to calculate the binding free energy through LIE approach. In this approach, the binding free energy is estimated from the change in the ele and vdW interaction energy between the ligand and its environment on complex formation [35]. The contributions of the interaction energy are determined from the snapshots of the MD simulations of free and complex-bound ligands in the solution by subtracting the energies of the individual components from the total energy of solvated systems [36]. To attain maximum accuracy, we calculated the energy contributions based on the averages of the conformational ensembles generated by the MD simulations of the complex and the free ligands in a solution and scaled individually [37]. For the bound ligand, we used the following:

$$E_{ele\;bound}=E_{ele\;total}-E_{receptor}+E_{ele\;water}-E_{ele\;ligand}$$ (3)

$$E_{vdW\;bound}=E_{vdW\;total}-E_{vdW\;receptor}+E_{vdW\;water}-E_{vdW\;ligand}$$ (4)

For the free ligand:

$$E_{ele\;free}=E_{ele\;total}-E_{ele\;ligand}-E_{ele\;water}$$ (5)

$$E_{vdW\;free}=E_{vdW\;total}-E_{vdW\;ligand}-E_{vdW\;water}$$ (6)

The binding free energy was estimated as the sum of the differences in the vdW and ele interaction energy scaled by weighting factors. The overall snapshots of the conformational ensemble were averaged as follows:

$$\Delta \;E_{LIE}=\alpha (E_{vdW\;bound}-E_{bdW\;free})+\beta (E_{ele\;bound}-E_{ele\;free})+\gamma$$ (7)

where α is 0.16, β is 0.5, and γ is 0. The differences in the ele and vdW interaction energy were calculated by a Perl program provided with FEW.

### 2.5 Computational alanine scanning

FoldX approach was used for computation alanine scanning [38], which was on the mutations of the binding site residues and the recalculations of the free energy for the mutated system based on the trajectories of complexes for each of the 10,000 snapshots along 200 ns simulation[39, 40]. For the FoldX calculations [41], a graphical user interface was utilized which was implemented as a plug-in for the YASARA molecular graphic suite [42]. If a free energy change occurred between a mutant and the WT proteins (active residues), namely, ΔΔG = ΔG (MT) − ΔG (WT) > 0, the mutation was considered destabilizing; otherwise, when ΔΔG < 0, the mutation was considered stabilizing. To identify the functionally important dynamic changes, we computed the average ΔΔG by selecting multiple samples (200–300) from the equilibrium ensembles in accordance with a modified FoldX protocol [43, 44].

## 3. Results and discussion

### 3.1 Quantum chemical calculations

**Fig 2:**
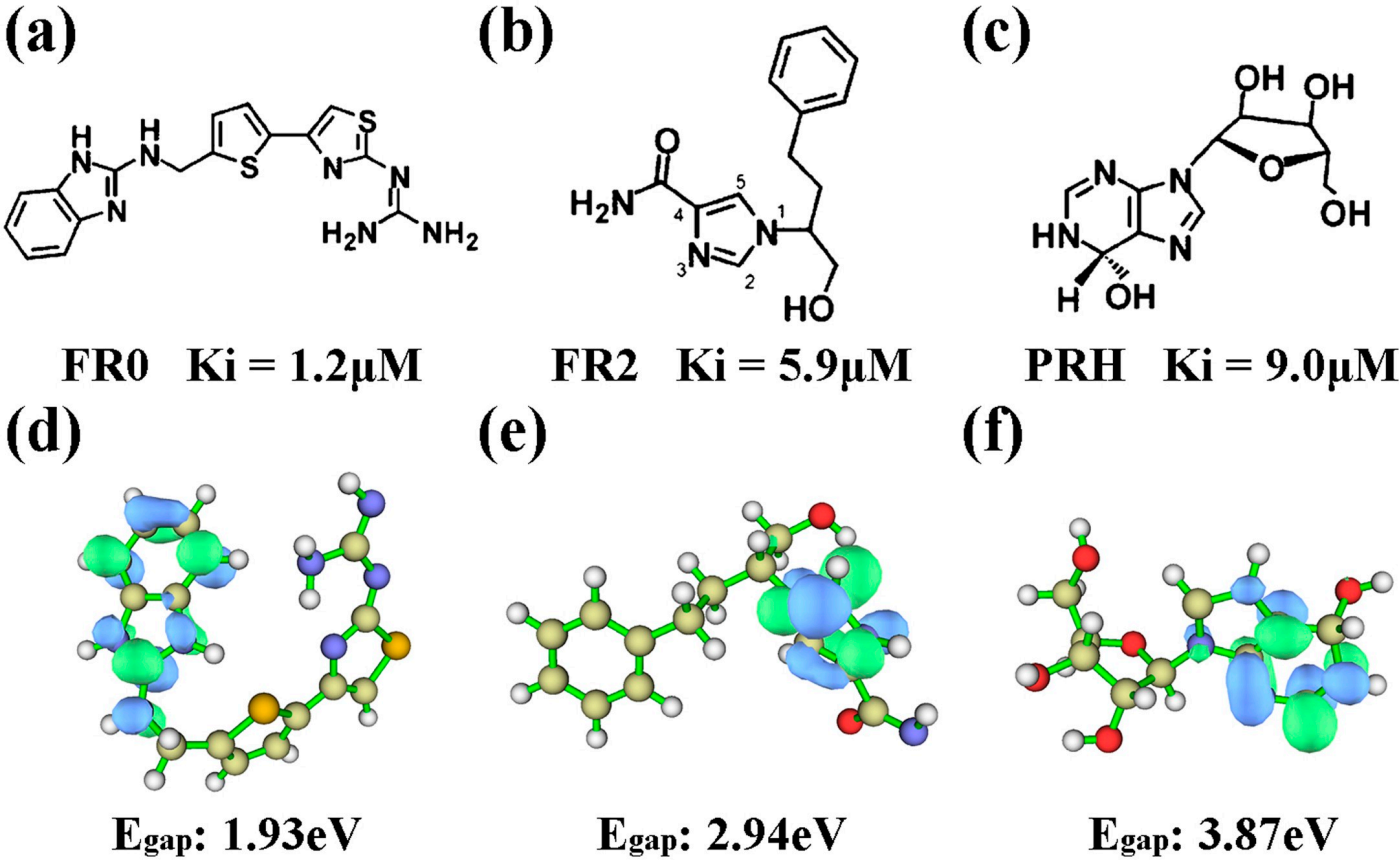
Chemical structures and LUMO orbits of the three ligands: FR0, FR2, and PRH. Ki and Egap of the three ligands are annotated.

Quantum chemical calculation was performed on the three ligands by using Gaussian09 [16] to optimize the structures of the three ligands and investigate their chemical characteristics. The energy difference between the HOMO and LUMO is termed the HOMO–LUMO gap. HOMO and LUMO are called frontier orbitals in frontier molecular orbital theory. The difference in energy between these two frontier orbitals can be used to predict the strength and stability of electron transfer [45]. The chemical structure and LUMO orbits of the three ligands are shown in Fig 2. Fig 2A–2C represented the inhibition constant (Ki) of the three compounds as determined by Takayoshi Kinoshita et al.[9]. FR0, FR2, and PRH had Ki 1.2, 5.9, and 9.0 μM, respectively. The inhibitory activities of the three compounds displayed the following trend: FR0 > FR2 > PRH. Fig 2D–2F show the LUMO orbits drawn by the Multiwfn program [46], representing the active part of the compound. Egap is the energy difference between HOMO and LUMO orbits [47]. This method considers all coordinates and provides a more efficient sampling approach than a geometrical reaction coordinate in reflecting the activities of compounds. In terms of Egap, the inhibitors followed the trend of FR0 < FR2 < PRH, indicating that electron transfer might occur more easily in FR0 than in FR2 and PRH. Thus, the ability of FR0 to form various interactions, such as H bonds, salt bridges, and vdW interactions, was stronger than that of FR2 and PRH. The comparison of Egap might be related to Egap of the inhibitory activities of the ligands. Those results were consistent with the research of Takashi in 2005 [9].

**Fig 3:**
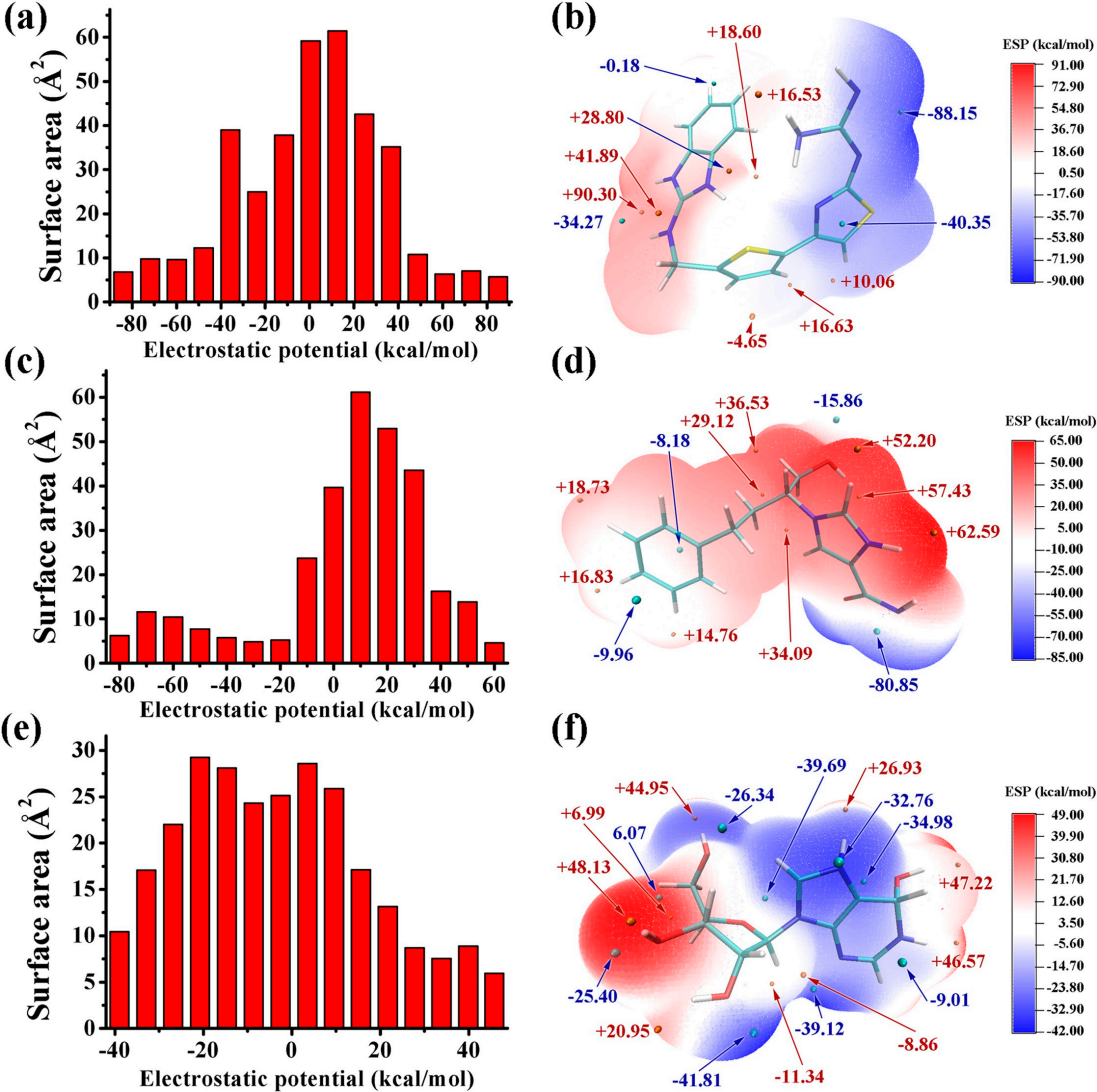
Electrostatic potential information of the three ligands: FR0, FR2, and PRH. Surface area in each electrostatic potential (ESP) ranges on the vdW surface of (a) FR0 (c) FR2 and (e) PRH. ESP-mapped molecular vdW surface of (b) FR0 (d) FR2 and (f) PRH.

The electrostatic potential (ESP) on the molecular vdW surface was calculated to investigate and predict intermolecular interactions. The result demonstrated that the strength and orientation of weak interactions, including H bonds and halogen bonds, can be predicted and explained by analyzing the magnitude and positions of the minima and maxima on the surface [48]. The ESP-mapped vdW surface and the surface extrema of the three ligands are shown in Fig 3A and 3C and 3E), and the surface areas of the ligands in their different ESP ranges are shown in Fig 3B, 3D and 3F). The values were 88.2 and 90.3 kcal•mol−1 for FR0, 80.1 and 62.6 kcal•mol−1 for FR2, and 41.9 and 45.0 kcal•mol−1 for PRH. The two amino groups of FR0, the imidazole and amino group of FR2, and the purine ring of PRH displayed the most negative charge in their respective structures, indicating that these parts were more likely to suffer the attack of the carboxyl group of Asp296. Terasaka [10] and Limongelli [11] found that Thr57-Ala73 and Ala183-Ile188 played important roles in the activity of ADA. The detailed interaction between ligand and ADA (S3 Fig) showed that those residues were also interacted with other inhibitors.

### 3.2 Structural stability of the five systems

**Fig 4:**
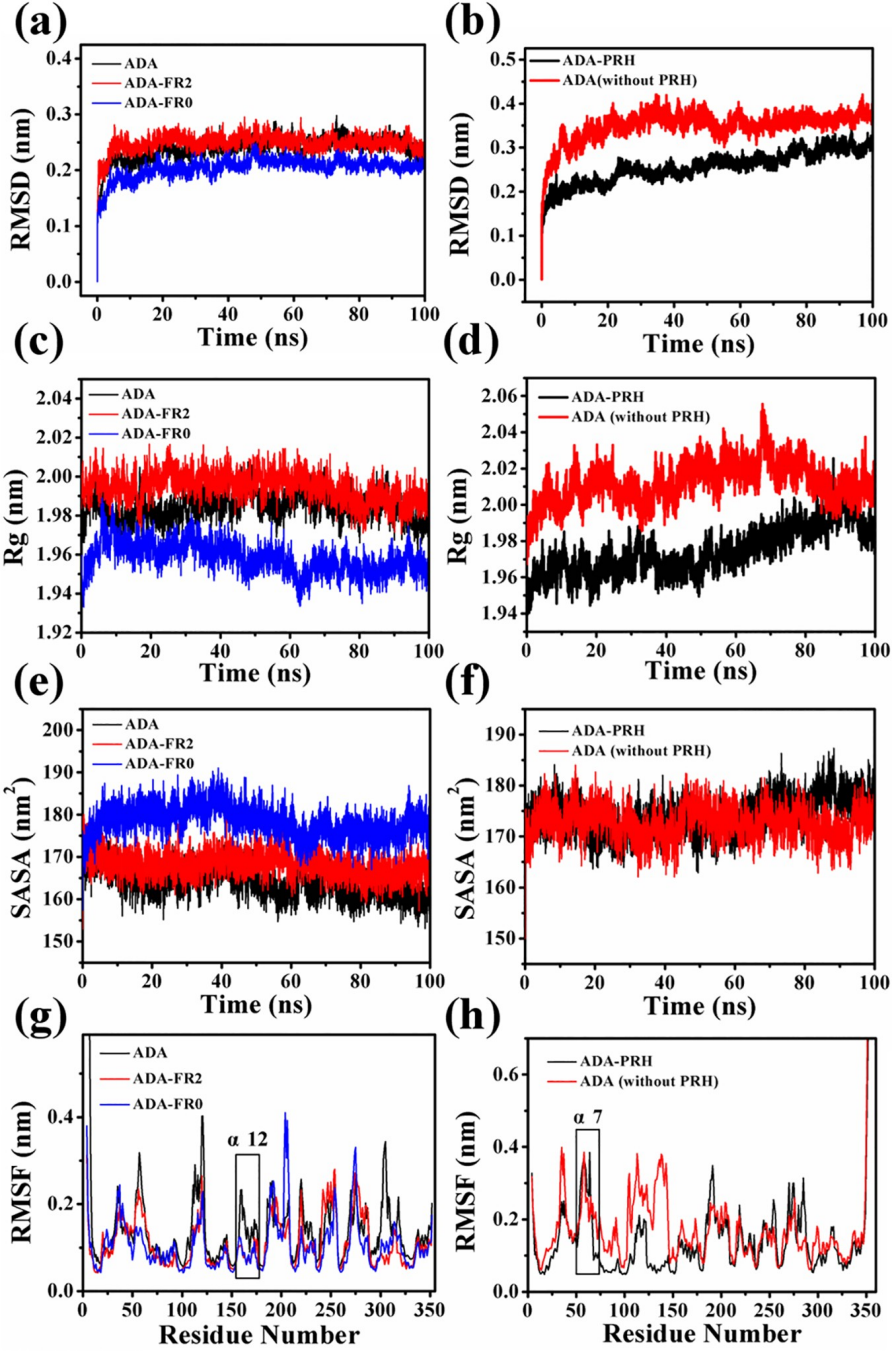
Structural stability of the five systems. (a) RMSD of C-α (c) radius of gyration (Rg) (e) SASA and (g) RMSF of three systems: ADA, ADA-FR2, and ADA-FR0. (b) RMSD of C-α (d) radius of gyration (f) SASA and (h) RMSF of the two systems: ADA-PRH and ADA (without PRH).

After 100 ns simulations, the root-mean-square deviations (RMSDs) of the Cα atom backbone of the five systems were calculated to evaluate whether the complex system could reach equilibrium during simulation. As shown in Fig 4A, the RMSDs of ADA and ADA-FR2 could be stabilized at 0.25 nm, and the RMSDs of ADA-FR0 could be stabilized at 0.2 nm after 5 ns, suggesting that the structures of the three systems already reached a state of relative equilibrium. The same findings can be observed in Fig 4B, that is, the RMSDs of ADA-PRH and ADA (without PRH) could be stabilized at approximately 0.25 and 0.35 nm, respectively, after 20 ns, suggesting that the structures of the two systems reached a state of relative equilibrium. The simulation was submitted twice, and the RMSDs of the second MD simulation are provided in S4 Fig. The radius of gyration (Rg) and SASA of the five systems were calculated by VMD program (Fig 4C–4F). As shown in Fig 4C, the Rg values of ADA and ADA-FR2 could be stabilized at 1.99 nm, and those of ADA-FR0 could be stabilized at 1.96 nm after 10 ns. In Fig 4D, the Rg values of ADA-PRH and ADA (without PRH) could be stabilized at 2.02 and 1.98 nm, could be stabilized at 170 nm2, and hoset of ADA-FR0 could be stabilized at 180 nm2 after 10 ns. In Fig 4F, the SASA values of ADA-PRH and ADA (without PRH) could be stabilized at 175 nm2. These two indices implied that the structures of the five systems classified to two groups reached stability and that their states attained equilibrium. The per-residue amino acid fluctuations of the five systems were investigated. The root-mean-square fluctuation (RMSF) values were calculated (Fig 4G and 4H). In Fig 4G, the α12 helix was highlighted because its RMSF was greater than that of ADA-FR2 and ADA-FR0. In Fig 4F, the α7 helix was highlighted, and both systems displayed large fluctuations. These two parts were close to the structural gate and the binding pocket, which would be examined in subsequent analysis.

### 3.3 Conformational changes caused by FR0 and FR2

**Fig 5:**
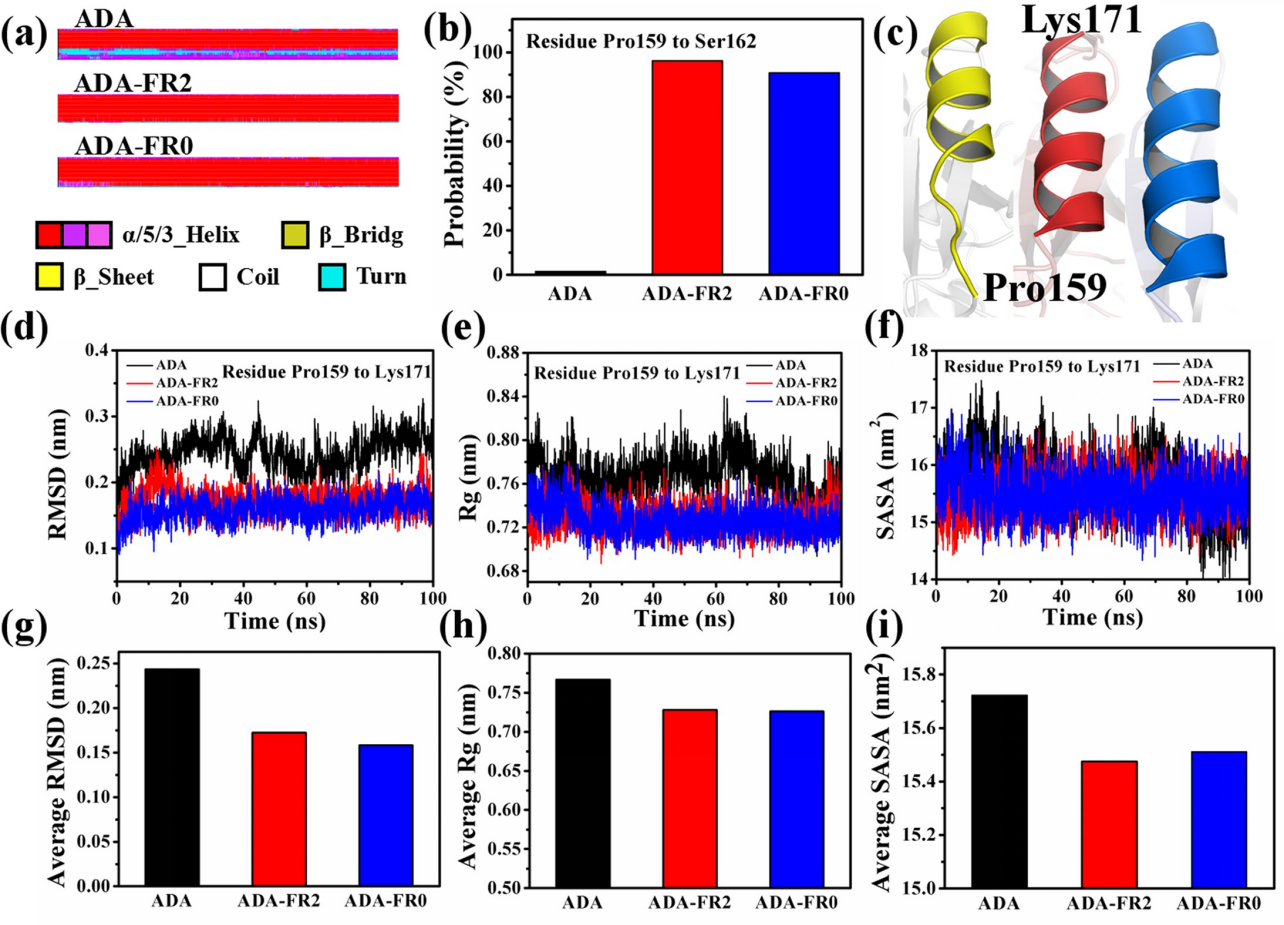
Conformation change in residues P159 to K171 of the three systems: ADA, ADA-FR2, and ADA-FR0. (a) Differences in the secondary structures of residues P150 to K171 of the three systems. (b) Probability of P159 to S162 to form a helix. (c) Structure of P159 to K171 (yellow for ADA, red for ADA-FR2, and blue for ADA-FR0). (d) RMSD; (e) Rg; and (f) SASA of P159 to K171 in the 100 ns simulations. (g) Average RMSD; (h) average Rg; and (i) average SASA of P159 to K171.

The conformational changes caused by FR0 and FR2 were investigated and compared with nonligated ADA because the ADAs of both inhibitors adopted an open form when they were bound. To investigate the conformational changes, we obtained the difference in the secondary structure (DSSP) by using the do_dssp command of the GROMACS 5.1.4 package [49]. The complete figure of the DSSP is shown in S5 Fig. The DSSP of residues Pro159 to Lys171 in ADA significantly differed from that in ADA-FR2 and ADA-FR0. In the figure depicting the DSSP, different colors represented various secondary structures; red and purple denote helixes, whereas blue indicates a turn. As shown in Fig 5A, residue Pro159 to Lys171 completely forms a helix in ADA-FR2 and ADA-FR0. However, these residues of ADA partly form a helix, and the left residues show a turn of structure. These residues formed the gate location of ADA[8]. To several residues (Pro159 to Ser162) depicted as blue in the DSSP, we calculated the probability to form a helix of the three systems (Fig 5B). The probabilities of residues Pro159 to Ser162 in ADA, ADA-FR2, and ADA-FR0 were 1.5%, 96.15%, and 90.78%, respectively. The probabilities of the four residues in ADA were considerably lower than those in the two other systems, suggesting that the α12 helix was likely to partly unfasten in the terminal of Pro159. The structure of Pro159 to Lys171 after 100 ns is depicted in Fig 5C to confirm this change, indicating the following: yellow for ADA, red for ADA-FR2, and blue for ADA-FR0. In comparison with the helix of α12 in ADA-FR2 and ADA-FR0, the helix of α12 was shortened in ADA, and the structure of several residues changed to a turn. The RMSD, Rg, and SASA values of residues Pro159 to Lys171 were calculated (Fig 5D–5F). In addition, the average RMSD, Rg, and SASA values were determined (Fig 5G–5I). The results of the three indices suggested that the fluctuation of residues Pro159 to Lys171 in ADA was greater than that in ADA-FR2 and ADA-FR0. Therefore, the binding of FR0 and FR2 was beneficial to maintaining the stability of the structure of ADA.

**Fig 6:**
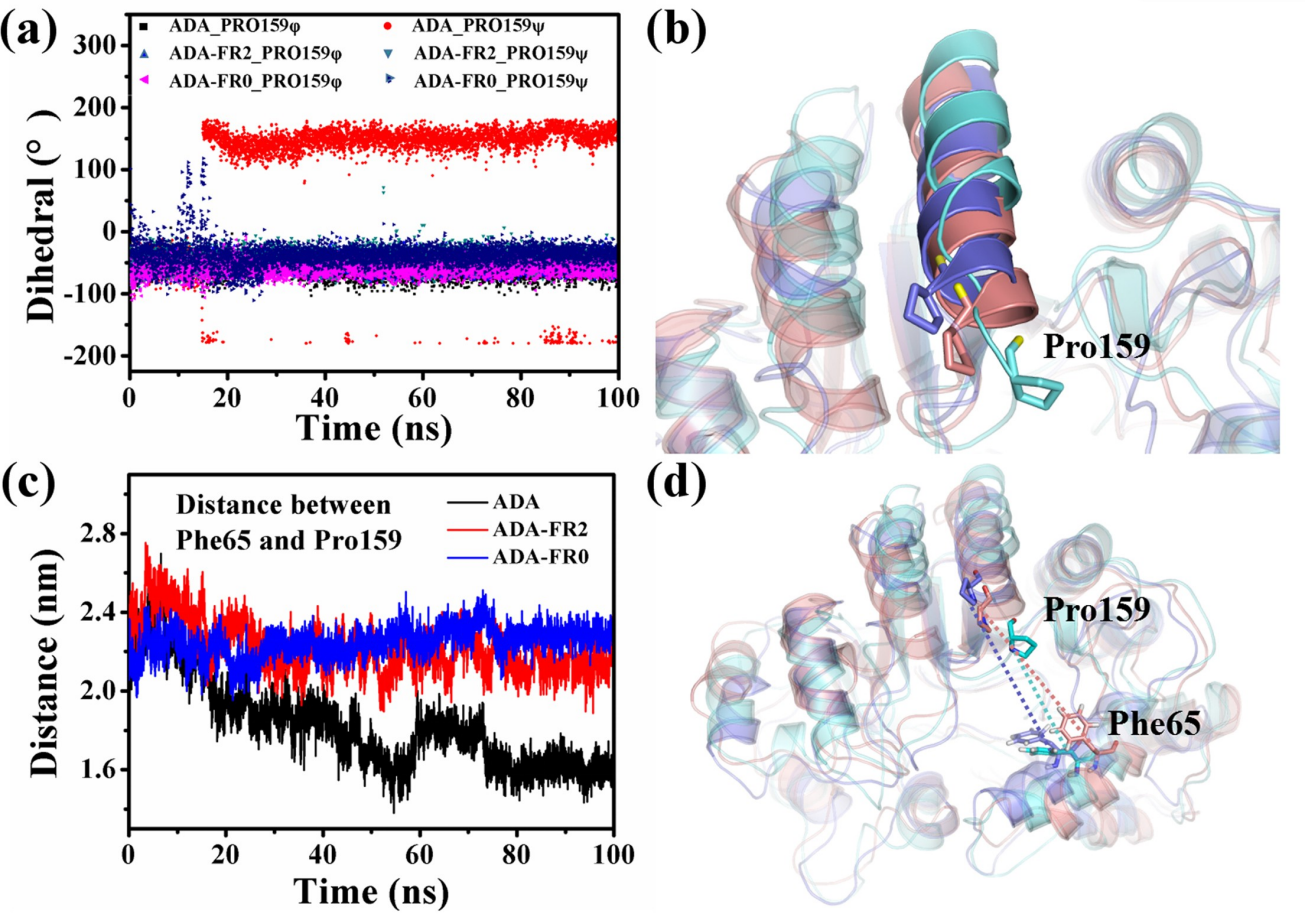
(a) Dihedral changes of Pro159 of the three systems. (b) Residues P159 to K171 are shown in cartoon, and Pro159 is shown in sticks. ADA is in cyan; ADA-FR2 is in salmon; and ADA-FR0 is in slate. (c) Distance changes between Phe65 and Pro159 of the three systems in the 100 ns simulations. (d) Distance between Phe65 and Pro159 of the three systems (ADA in cyan, ADA-FR2 in salmon, and ADA-FR0 in slate).

To investigate the influence of the conformational changes in the binding pocket of ADA, we monitored the changes in the dihedral angle of Pro159 of the three systems. Fig 6A shows the dihedrals of Pro159 of the three systems in the 100 ns simulations. The φ of ADA Pro159 changed significantly, whereas the other dihedrals maintained a specific angle. To observe this change intuitively, we depicted the structure of ADA with a cartoon and showed Pro159 with a stick (Fig 6B). The angle and position of ADA Pro159 changed substantially, whereas these parameters in the two other systems were almost immovable. The binding pocket may be slightly changed because of the change in the position of residue Pro159. The distance between Pro159 and Phe65 (Fig 6D) in the 100 ns simulations was calculated (Fig 6C). In ADA, the distance decreased immediately at the onset of the simulation and stabilized at approximately 1.7 nm, whereas the distances in ADA-FR2 and ADA-FR0 were almost unchanged and stabilized at approximately 2.2 nm. The difference in the distance in ADA and the two systems reflects the movement of Pro159, possibly leading to the reduction of the binding pocket because two key residue fragments were relatively moved. In summary, the binding of FR0 and FR2 could maintain the conformation of the binding pocket and the stability of the structure of ADA.

### 3.4 Conformational changes caused by PRH

**Fig 7:**
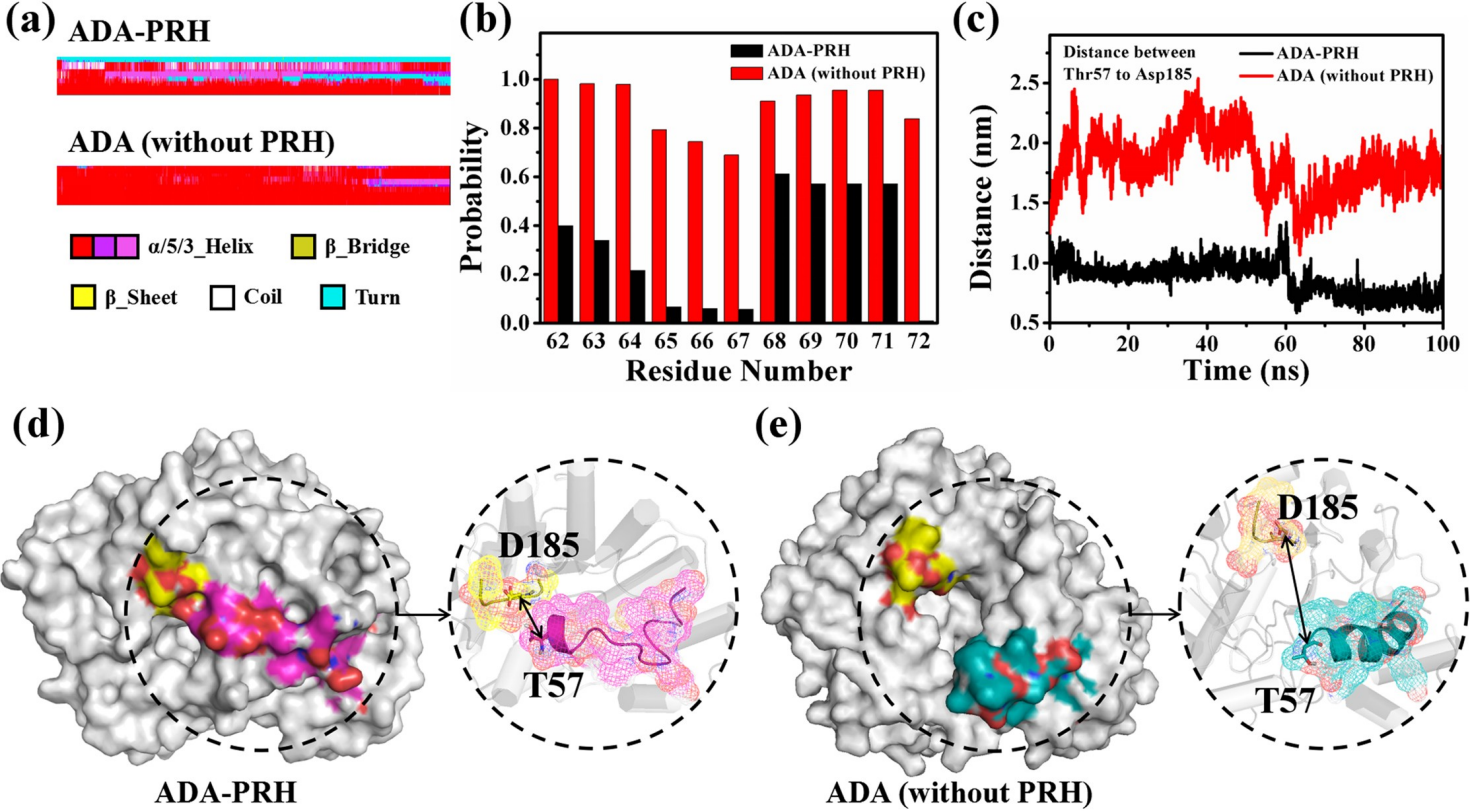
Conformation change in residues T57 to A73 of the two systems: ADA-PRH and ADA (without PRH). (a) Differences in the secondary structures (DSSPs) of T57 to A73 between the two systems. (b) Probabilities of L62 to I72 to form a helix. (c) Distance changes between Thr57 and Ile185 in the 100 ns simulations. (d) ADA-PRH complex (left) on the surface, residues T57 to A73, and residues A183 to I188 (right) in a mesh. (e) ADA (without PRH) (left) on the surface, residues T57 to A73, and residues A183 to I188 (right) in a mesh.

The conformational changes caused by inhibitor PRH in the ADA-PRH complex were compared with that in ADA (without PRH) by considering the same sequence of ADA. The only difference of the two systems is the presence of the ligand PRH. When binding to PRH, ADA adopts a closed form. Therefore, we explored whether ADA still adopted a closed form when PRH was removed from the ADA-PRH complex. First, the DSSPs of the two systems were obtained (S6 Fig). The RMSF values and the position of the residues indicated that the α7 helixes in ADA-PRH and ADA (without PRH) were considerably different. In Fig 7A, for residues Thr57 to Ala73, the secondary structure showed that a part of the residues formed a helix, and the other part of the residues generated a turn in ADA-PRH. By contrast, all of the residues created a helix in ADA (without PRH). The probability of residues Leu62 to Ile72 to form a helix was calculated (Fig 7B). The probability of residues Leu62 to Ile72 in ADA (without PRH) was almost above 80%, whereas their probability in ADA-PRH was almost lower than 50%. The probability of ADA-PRH was lower than that of ADA (without PRH), indicating that the α7 helix was mostly destroyed in ADA-PRH compared with that of ADA (without PRH). To facilitate the comparison, we showed the structures of ADA by using the conformation in 100 ns for ADA-PRH and ADA (without PRH) (Fig 7D and 7E, respectively). In the left panel of Fig 7D and 7E, ADA is shown as the surface, residues Thr57 to Ala73 are highlighted in magenta for ADA-PRH and green for ADA (without PRH), and residues Ala183 to Ile188 are highlighted in yellow. In Fig 7D, the structural gate, which consisted of residues Thr57 to Ala73 and residues Ala183 to Ile188, maintained a closed state, suggesting that ADA in ADA-PRH also adopted a closed form after the 100 ns simulations. However, the structural gate changed to an open form, suggesting that if PRH was removed from the ADA-PRH complex, ADA would adopt an open form after the MD simulations (Fig 7E). More detailed structures of the two systems are presented in the right panel of Fig 7D and 7E, and the color scheme is the same as that in the left panel. In addition, these two parts of the amino acid residues are displayed as mesh, and residues Thr57 and Asp185 are shown as sticks. The changes in the distance between the two parts of the amino acid residues comprising the structural gate and the structural changes in the α7 helix were observed. Therefore, the distance between Thr57 and Asp185 during the 100 ns simulations was monitored. In Fig 7C, the distance increased rapidly and stabilized at approximately 2 nm in ADA-PRH, whereas the distance remained stable at approximately 1 nm in ADA (without PRH). Therefore, the structural gate changed to an open form in ADA (without PRH).

**Fig 8:**
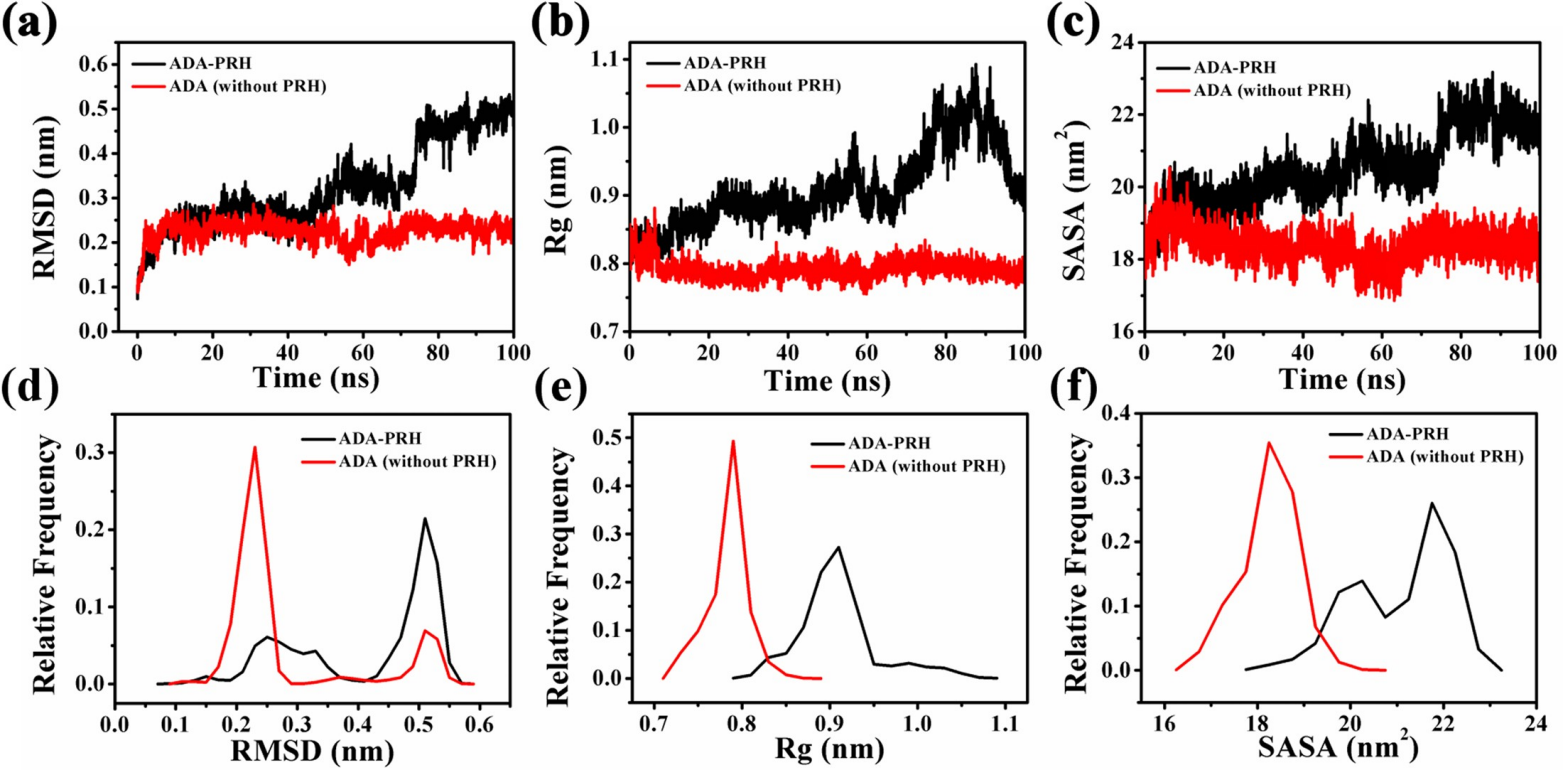
Structure stability of T57 to A73 of the two systems. (a) RMSD; (b) Rg; and (c) SASA of residues T57 to A73 in the 100 ns simulations. (d) Relative frequencies of RMSD; (e) Rg; and (f) SASA of residues T57 to A73.

**Fig 9:**
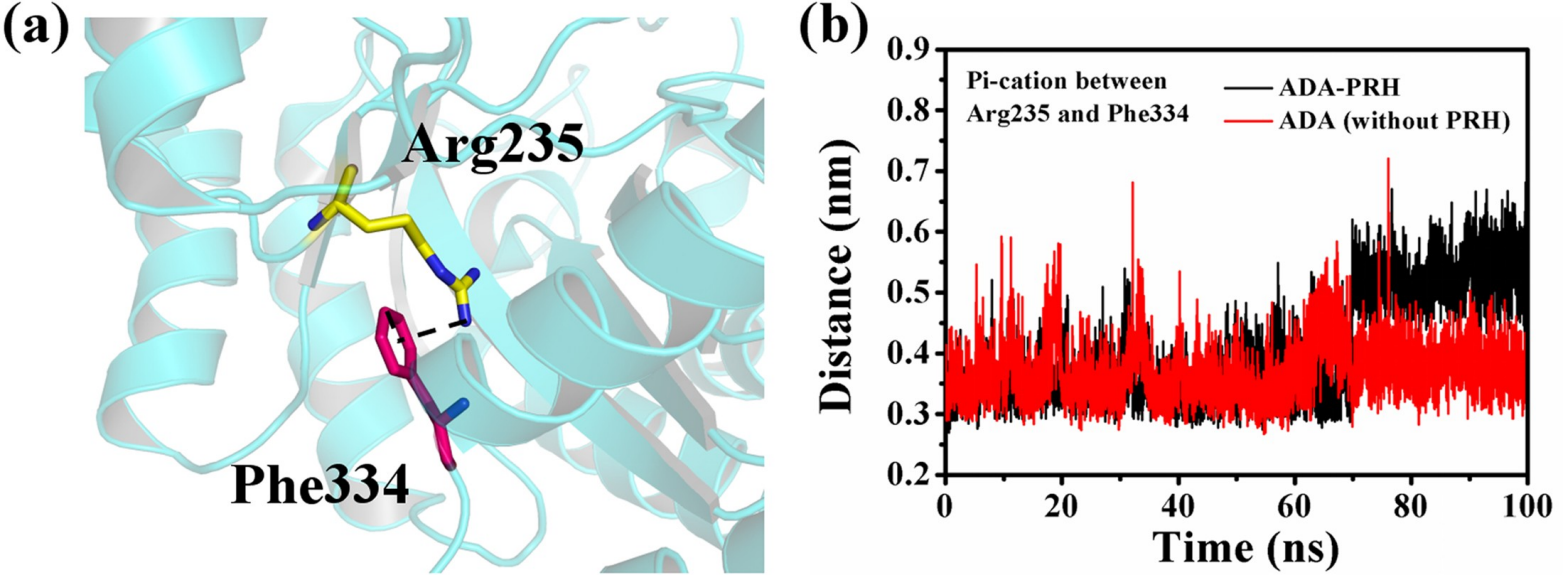
Cation–pi interaction between Arg235 and Phe334. (a) Arg235 is depicted in a yellow stick; and Phe334 is shown in a magenta stick. (b) The distance between the NH1 atom of Arg235 and the center of the benzene group of Phe334 in the 100 ns simulations.

RMSD, Rg, and SASA and the relative frequency of residues Thr57 to Ala73 were also calculated (Fig 8). The changes in the three indices indicated that the structure of residues Thr57 to Ala73 in ADA-PRH changed substantially compared with that in ADA (without PRH). To further investigate the influence of the binding of PRH to the structure of ADA, we examined the cation–pi interaction between Arg235 and Phe334 and calculated the distance between the NH1 atom of Arg235 and the center of the benzene group of Phe334 in the 100 ns simulations (Fig 9). In Fig 9A, the cation–pi interaction existing between residues Arg235 and Phe334 is denoted by a black dash, and the two residues are shown as sticks. In Fig 9B, the distances between the NH1 atom of Arg235 and the center of the benzene group of Phe334 of the two systems were substantially different in the 100 ns simulations. The distance in ADA-PRH increased from 0.35 nm to 0.55 nm and stabilized during the simulations, whereas the distance in ADA (without PRH) was stabilized at approximately 0.35 nm from the beginning to the end of the simulations. By contrast, the cation–pi interaction weakened and even disappeared in ADA-PRH. The cation–pi interaction is beneficial to the stability of protein structures. Therefore, this change in the cation–pi interaction indicated that the binding of PRH destroyed the structural stability of the original ADA. Thus, when binding to PRH, the α7 helix was greatly unfastened, and the relative opposition between Thr57–Ala73 and Ala183–Ile188 was closed, causing ADA to adopt a closed form. If PRH was removed from the ADA-PRH complex, the structural gate opened, and ADA adopted an open form. The binding of PRH could not maintain the stability of original ADA structure. In order to show more clearly the effect of PRH on ADA conformation, we created animations about simulate trajectories (Supplementary material). It can be clearly seen that losing PRH could turn the ADA from the closed form to the open open.

### 3.5 PCA, H bond analysis, and binding free energy calculations

**Fig 10:**
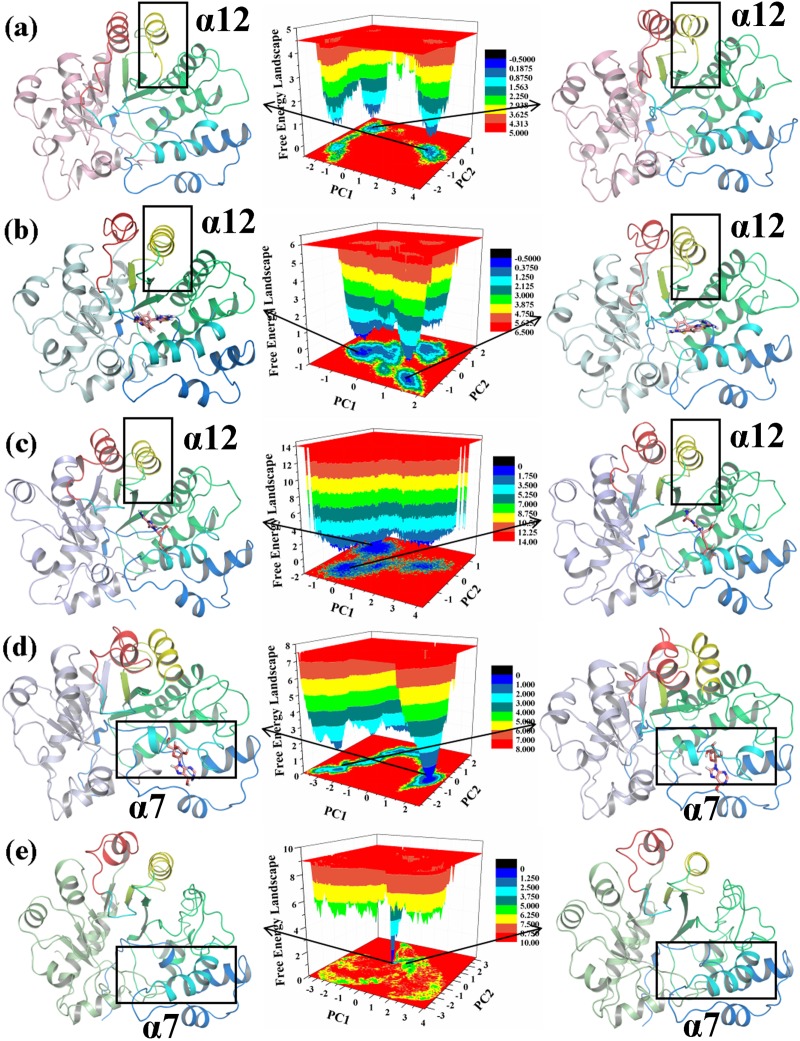
Free energy landscape (FEL) and structures of the two most stable structures of the five systems. The α7 helix is colored cyan; the structural gate is colored cyan, which consist of the α7 helix (T57-A73) and residues A183 to I188. (a) ADA. (b) ADA-FR0 complex. (c) ADA-FR2 complex. (d) ADA-PRH complex. (e) ADA (without PRH).

To confirm whether the conformational changes were continuous and stable, we performed PCA. The FEL is drawn in Fig 10. In the FEL of the five systems located in the middle panel of Fig 10, different energies of the corresponding structure are shown in different colors. The conformations found in the blue area are more stable and have lower energy states than those found in the red area [50]. Table 1 lists the probabilities of PC1 and PC2 of the three systems that were obtained by PCA. The two most stable conformations of the ADA structure in the five systems are shown in the left and right panels of Fig 10. The α12 helixes (residues Pro159 to Lys171) in ADA, ADA-FR2, and ADA-FR0 (yellow) and the α7 helixes (residues Thr57 to Ala73) in ADA-PRH and ADA (without PRH) (cyan) are highlighted. The structures of the two most stable conformations of ADA revealed that the conformational changes in the α12 helix existed between ADA, ADA-FR2, and ADA-FR0. This finding was consistent with the previous analysis. The conformational changes in α7 between ADA-PRH and ADA (without PRH) were also consistent with previous analysis. Therefore, the conformational changes were continuous and stable, and the previous analysis was reliable.

**Table 1:** The probability of PC1 and PC2 during MD simulation.

System	Principal Component (PC)	Probability
ADA	PC1	54.33%
	PC2	7.39%
ADA-FR0	PC1	18.09%
	PC2	11.9%
ADA-FR2	PC1	28.1%
	PC2	8.79%
ADA-PRH	PC1	32.47%
	PC2	11.67%
ADA (without PRH)	PC1	32.01%
	PC2	14.1%

To further compare the inhibitory activities of the three ligands, we calculated the H bond between ADA and the ligand in the three ligated systems (ADA-FR0, ADA-FR2, and ADA-PRH). The donor and acceptor of the H bond and the probability of the H bond in the 100 ns simulation are listed in Tables 2–4. To attain more accurate statistical results, we selected only the H bonds with a probability greater than 20%. The comparison of these tables showed that the number of H bonds differed in the three systems. ADA-FR0 was the most, whereas ADA-PRH was the least. This trend corresponded to Ki of the three ligands. The key residues of the binding of the inhibitors were His17, Asp19, Phe65, Met69, Leu106, Trp117, Met155, Gly184, Asp295, and Asp296. Among them, four residues (His17, Asp19, Phe65, and Asp296) were critical to the binding.

**Table 2:** The probability of H-bond between ADA and FR0 during MD simulation.

Donor	Acceptor	Probability
FR0-C23	ASP19-OD2	53.43%
FR0-C23	ASP19-OD1	48.55%
FR0-C23	ASP296-OD2	46.10%
FR0-C17	HIS17-NE2	45.75%
FR0-N29	TRP117-CH2	41.73%
FR0-N26	PHE65-CD1	30.08%
FR0-N28	MET155-CE	28.87%
FR0-N26	MET69-SD	27.24%
FR0-N29	TRP117-CZ3	24.59%
FR0-C18	HIS17-NE2	24.45%
FR0-C17	HIS17-CE1	22.94%
FR0-N29	LEU106-CD1	22.23%

**Table 3:** The probability of H-bond between ADA and FR2 during MD simulation.

Donor	Acceptor	Probability
FR2-O15	MET69-SD	38.27%
FR2-C1	ASP19-OD1	30.27%
FR2-O15	PHE65-CZ	27.23%
FR2-O15	ASP19-Main-O	27.13%
FR2-C1	ASP19-OD2	26.02%
FR2-O15	MET69-CE	23.21%
FR2-N10	MET155-CE	22.25%
FR2-O15	LEU106-CD1	21.40%

**Table 4:** The probability of H-bond between ADA and PRH during MD simulation.

Donor	Acceptor	Probability
PRH-C8	HIS17-ND1	42.70%
PRH-C8	HIS17-CG	37.43%
PRH-O6	ASP295-OD2	33.52%
PRH-C8	ASP296-OD2	26.68%
GLY181-Main-N	PRH-N3	21.99%
PRH-C8	ASP296-OD1	21.15%

**Fig 11:**
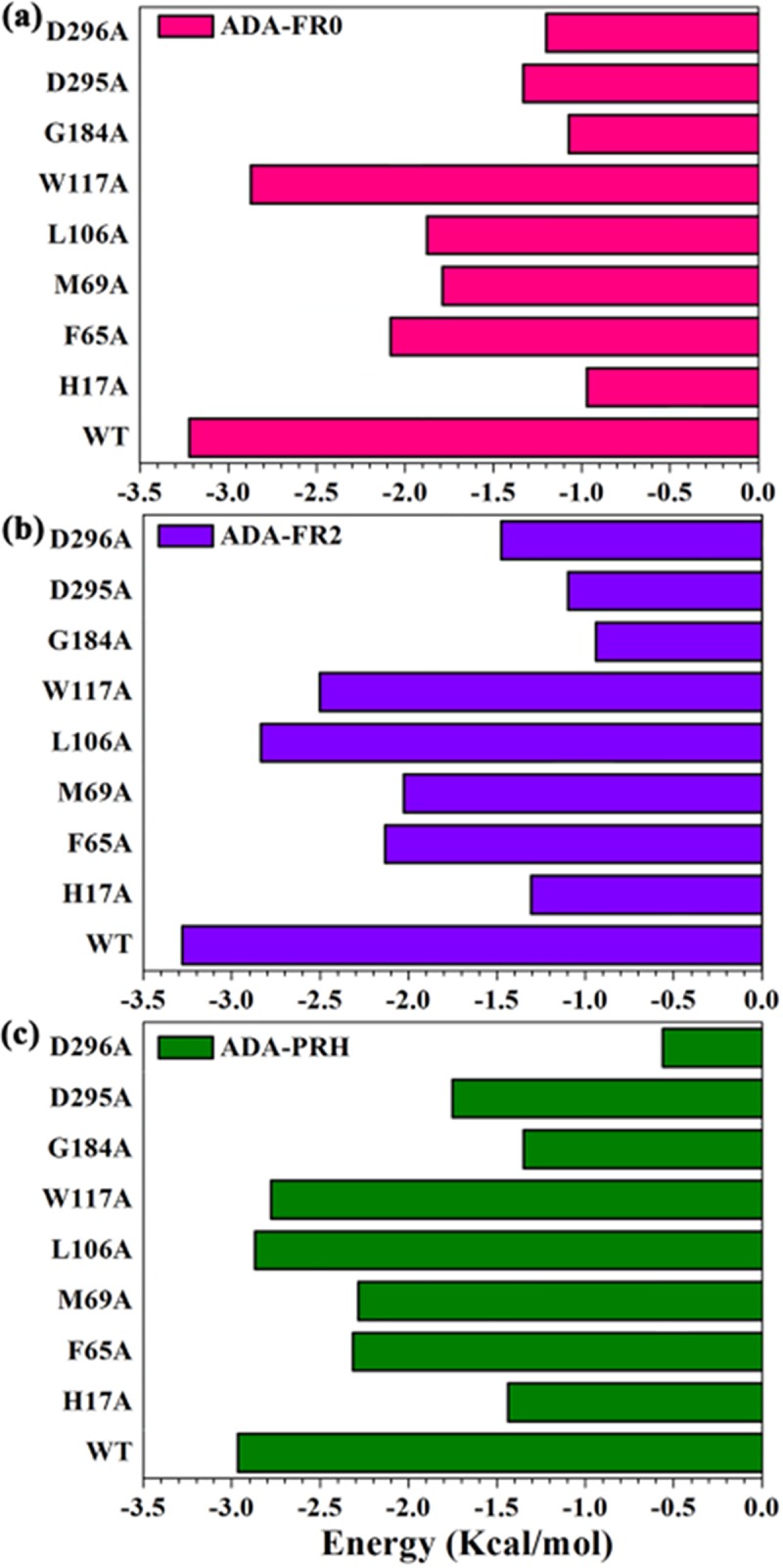
Computational alanine scanning of the binding site residues in open and closed inhibitor complexes. Analysis was performed using FoldX approach on the conformational ensembles obtained from the 200 ns MD simulations. Binding free energies and alanine scanning of the binding site residues are shown for ADA-FR0 α-ZOL (a), ADA-FR2 (b), and ADA-PRH (c). Energetic binding hotspots correspond to residues whose alanine scanning results indicate a significant decrease in binding free energy.

Computational alanine scanning of the selected amino acid residues of the inhibitor binding site groove was performed to elucidate the role of individual residues in the stability of protein structures. In this analysis, the residues surrounding the inhibitors (FR0, FR2, and PRH) were mutated to alanine to detect their influence on the stability of protein structures. The results of mutagenesis are presented in Fig 11. As shown, the mutation of the binding residues with inhibitors reduced the stability of ADA. Among them, mutations in His17, Gly184, Asp295, and Asp296 exerted the greatest effects on the protein energy, indicating that they played crucial roles in binding to inhibitors.

The binding free energies of the three ADA complex systems were calculated by LIE method. The delta ele (dE-ele), the delta vdW energy (dE-vdW), and the binding free energy estimate (dG-bind) was calculated (Table 5). dE-ele represents the difference between Eele bound and Eele free. dE-vdW denotes the difference between EvdW bound and EvdW free. The estimate of the binding free energy (dG-bind) was calculated using the equation dG-bind = α × dE-vdW + β × dE-ele with fixed coefficients of α = 0.16 and β = 0.5. The total binding free energies were −29.74, −28.47, and −18.28 kcal/mol for ADA-FR0, ADA-FR2, and ADA-PRH, respectively. The values of the binding free energies displayed the following trend: ADA-FR0 < ADA-FR2 < ADA-PRH. In general, the structure of a complex with a low binding free energy is relatively stable. Therefore, the structure of the ADA-FR0 complex was more stable than that of the ADA-FR2 complex, which in turn was more stable than that of the ADA-PRH complex. The conclusion that FR0 and FR2 were more beneficial than PRH to the stability of the ADA structure was confirmed by the binding free energy calculations. The comparison of the binding free energies supported the comparison of the inhibitory activities of the three inhibitors.

**Table 5:** Average van der Waals, electrostatic interaction energies and binding free energies between inhibitors and ADA.

	dE-vdW (kcal/mol)	dE-ele (kcal/mol)	dG-bind (kcal/mol)
ADA-FR0	-38.83	-43.46	-29.74
ADA-FR2	-35.90	-45.46	-28.47
ADA-PRH	-26.45	-28.10	-18.28

## 4. Conclusion

ADA catalyzes the deamination of adenosine and plays an important role in purine metabolism. In this study, five systems, namely, ADA, ADA-FR0, ADA-FR2, ADA-PRH, and ADA (without PRH), were subjected to 100 ns MD simulations. The systems were classified into two groups based on their sequences. The first group included ADA, ADA-FR0, and ADA-FR2. The second group comprised ADA-PRH and ADA (without PRH). The chemical structures of the three ligands were optimized, and the chemical characteristics of the three ligands were explored by using Gaussian09 [16]. The analysis of Egap and ESP on the molecular vdW surface might be related to the comparison of the inhibitory activities of the three ligands. After 100 ns simulations, the conformational changes in the five systems were investigated by using various methods and programs. In the open form, the probability of residues Pro159 to Lys171 of ADA-FR0 and ADA-FR2 to form a helix moderately increased compared with that in the nonligated ADA, and the binding of FR0 and FR2 was beneficial to the stability of the structure of ADA. In the closed form, when the PRH inhibitor was removed from the ADA-PRH complex, the structural gate opened, and the conformation of ADA changed to an open form. In comparison with the open form, the α7 (residues Thr57 to Ala73) of ADA with the closed form was mostly unfastened, and the stability of the original structure of ADA decreased because of the effect of PRH. The analyses of the H bond and the binding free energy supported the comparison of the inhibitory activities of the three ligands, thereby showing the trend of FR0 > FR2 > PRH based on the number and probability of H bonds and the relative superiority of the binding free energy. This study provides a theoretical basis for the mechanism and development of new ADA inhibitors.

## Supporting information

S1 Fig Overview of open form (PDB code: 1VFL) and close form (PDB code: 1KRM). (a) Open form. The structural gate is consist of residue T57 to A73 (in yellow) and residue A183 to I188 (in blue). (b) Close form. The structural gate is consist of residue T57 to A73 (in yellow) and residue A183 to I188 (in red). (c) The binding pocket of open form, which is consist of S0 subunit, F0 subunit, and additional subunits F1 and F2. (d) The binding pocket of close form, which consist of the subunit of S0 and F0.
(PDF)

S2 Fig Sequence alignment of the four proteins: 1VFL, 1NDW, 1NDV and 1KRM. (PDF)

S3 Fig The detailed interactions between ligand and ADA. (PDF)

S4 Fig The RMSD of C-α of the second time MD simulation. (a) RMSD of ADA, ADA-FR0 and ADA-FR2; (b) RMSD of ADA-PRH and ADA (without PRH).
(PDF)

S5 Fig The difference of secondary structure of ADA in the three systems: ADA, ADA-FR2 and ADA-FR0. (PDF)

S6 Fig The difference of secondary structure of ADA in the two systems: ADA-PRH and ADA (without PRH). (PDF)

S1 Table Composition of five systems. (PDF)
