# Supplementary material for: Probing inhibition mechanisms of adenosine deaminase by using molecular dynamics simulations
Source: PLoS One. 2018 Nov 16;13(11):e0207234. doi: 10.1371/journal.pone.0207234 (PMC6239307; doi:10.1371/journal.pone.0207234)

S1 Fig. Overview of open form (PDB code: 1VFL) and close form (PDB code: 1KRM). (a) Open form. The structural gate is consist of residue T57 to A73 (in yellow) and residue A183 to I188 (in blue). (b) Close form. The structural gate is consist of residue T57 to A73 (in yellow) and residue A183 to I188 (in red). (c) The binding pocket of open form, which is consist of S0 subunit, F0 subunit, and additional subunits F1 and F2. (d) The binding pocket of close form, which consist of the subunit of S0 and F0.

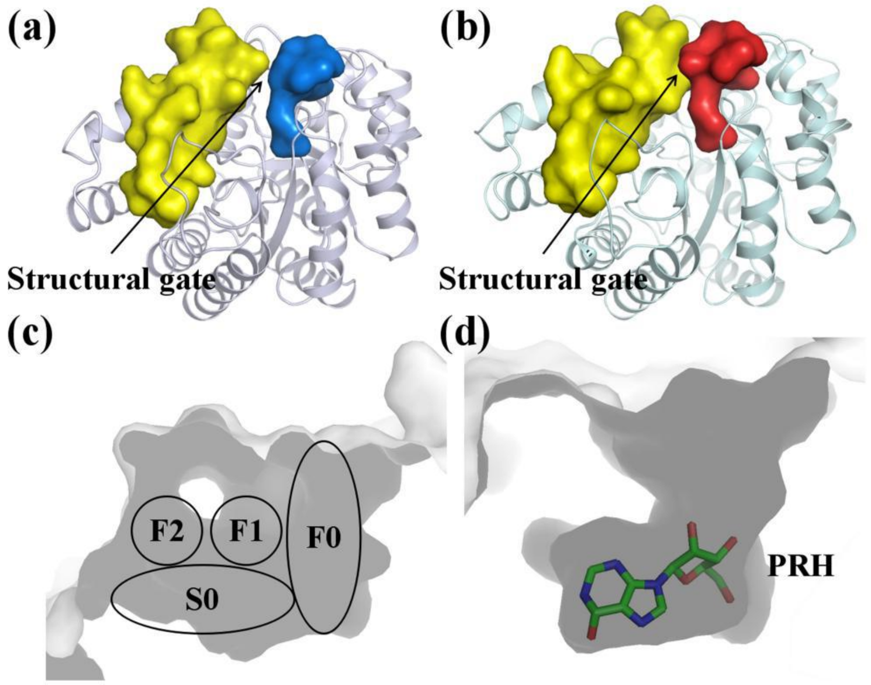

Supplement: S1 Fig — (a) Open form. The structural gate is consist of residue T57 to A73 (in yellow) and residue A183 to I188 (in blue). (b) Close form. The structural gate is consist of residue T57 to A73 (in yellow) and residue A183 to I188 (in red). (c) The binding pocket of open form, which is consist of S0 subunit, F0 subunit, and additional subunits F1 and F2. (d) The binding pocket of close form, which consist of the subunit of S0 and F0. (PDF) [file pone.0207234.s001.pdf]
