# Supplementary material for: Probing inhibition mechanisms of adenosine deaminase by using molecular dynamics simulations
Source: PLoS One. 2018 Nov 16;13(11):e0207234. doi: 10.1371/journal.pone.0207234 (PMC6239307; doi:10.1371/journal.pone.0207234)

S2 Fig. Sequence alignment of the four proteins: 1VFL, 1NDW, 1NDV and 1KRM.

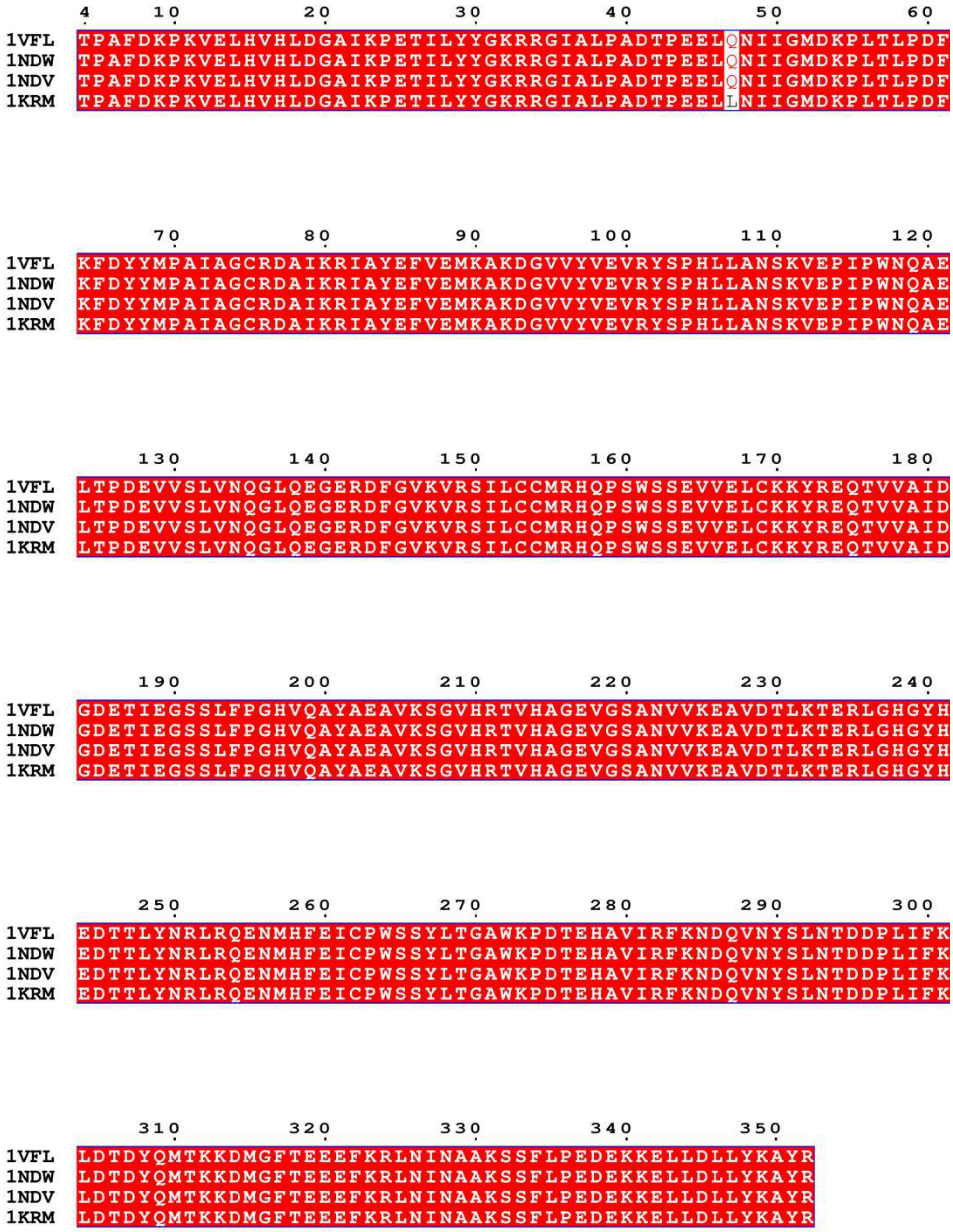

Supplement: S2 Fig — (PDF) [file pone.0207234.s002.pdf]
