# Supplementary figures and images for: Probing inhibition mechanisms of adenosine deaminase by using molecular dynamics simulations
Source: PLoS One. 2018 Nov 16;13(11):e0207234. doi: 10.1371/journal.pone.0207234 (PMC6239307; doi:10.1371/journal.pone.0207234)

**S3 Fig. The detailed interactions between ligand and ADA.**

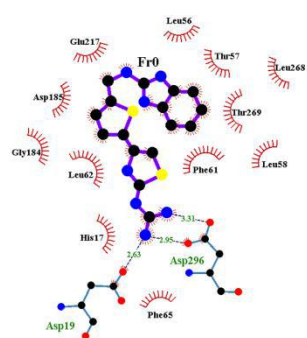

**1ndv**

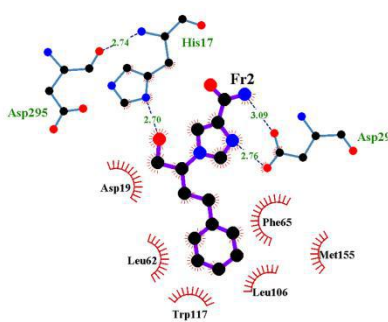

**1ndw**

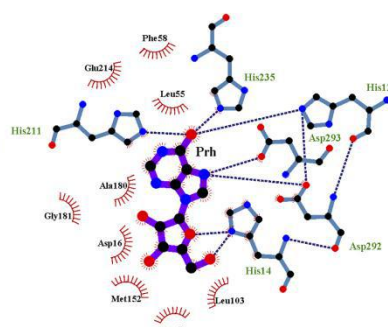

**1krm**

Supplement: S3 Fig — (PDF) [file pone.0207234.s003.pdf]

**S5 Fig.**The difference of secondary structure of ADA in the three systems: ADA, ADA-FR2 and ADA-FR0.

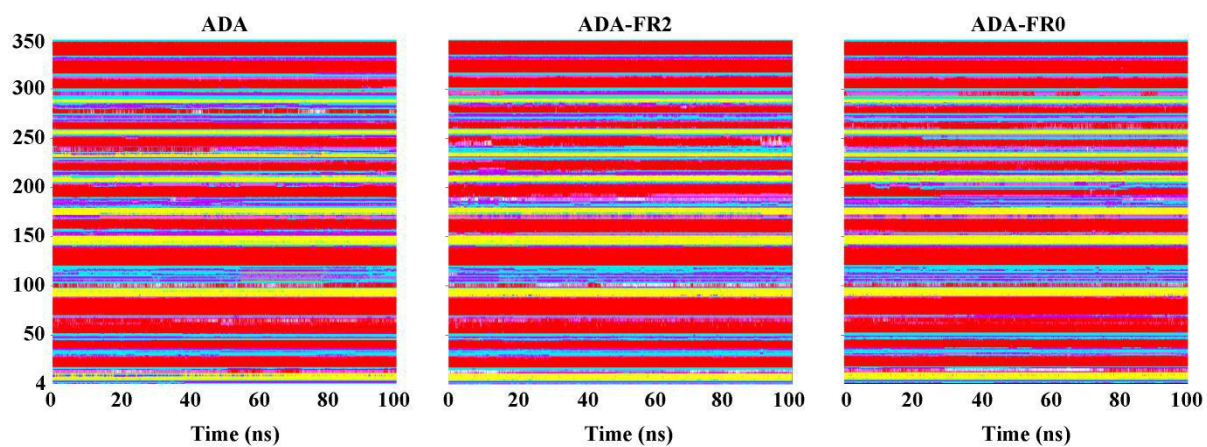

Supplement: S5 Fig — (PDF) [file pone.0207234.s005.pdf]

**S6 Fig. The difference of secondary structure of ADA in the two systems: ADA-PRH and ADA (without PRH).**

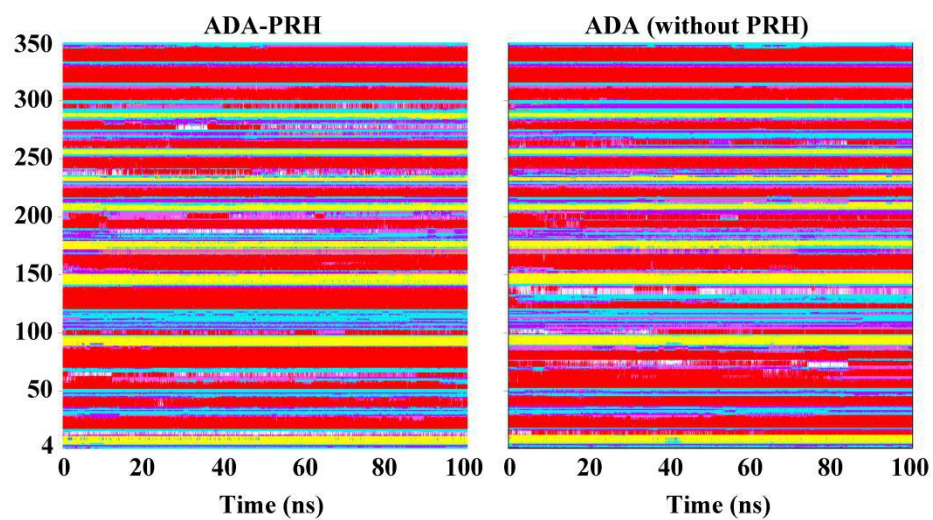

Supplement: S6 Fig — (PDF) [file pone.0207234.s006.pdf]
