# Supplementary material for: Probing inhibition mechanisms of adenosine deaminase by using molecular dynamics simulations
Source: PLoS One. 2018 Nov 16;13(11):e0207234. doi: 10.1371/journal.pone.0207234 (PMC6239307; doi:10.1371/journal.pone.0207234)

S4 Fig.The RMSD of C-α of the second time MD simulation. (a) RMSD of ADA, ADA-FR0 and ADA-FR2; (b) RMSD of ADA-PRH and ADA (without PRH).

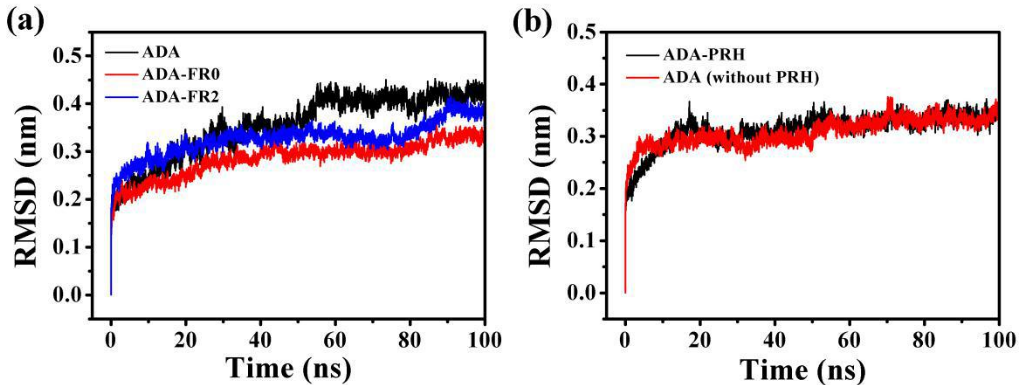

Supplement: S4 Fig — (a) RMSD of ADA, ADA-FR0 and ADA-FR2; (b) RMSD of ADA-PRH and ADA (without PRH). (PDF) [file pone.0207234.s004.pdf]
