# Supplementary material for: Probing inhibition mechanisms of adenosine deaminase by using molecular dynamics simulations
Source: PLoS One. 2018 Nov 16;13(11):e0207234. doi: 10.1371/journal.pone.0207234 (PMC6239307; doi:10.1371/journal.pone.0207234)
Supplement: S1 Table — (PDF) [file pone.0207234.s007.pdf]

S1 Table. Composition of five systems.

System	Protein	Ligand	Ions	SOL
ADA	1	None	Na (9)	14280
ADA-FR0	1	FR0	Na (10)	10555
ADA-FR2	1	FR2	Na (10)	10916
ADA-PRH	1	PRH	Na (11)	9654
ADA (without PRH)	1	None	Na (11)	14475
